# A Rare Diagnosis of Parotid Gland Follicular Lymphoma Arising in Warthin Tumor: Case Report and Literature Review

**DOI:** 10.3390/medicina60122086

**Published:** 2024-12-19

**Authors:** Ido Vaknin, Irit Allon, Shirley Zafrir-Haver, Alex Abramson

**Affiliations:** 1 Oral and Maxillofacial Surgery Department, Barzilai University Medical Center, Ben Gurion University of the Negev, Beer Sheva 8443944, Israel; idovaknin23@gmail.com; 2 Institute of Pathology, Barzilai University Medical Center, Ashkelon 7830604, Israel; 3 Faculty of Health Sciences, Ben Gurion University of the Negev, Beer Sheva 8443944, Israel; 4 Department of Hematology, Barzilai University Medical Center, Ben Gurion University of the Negev, Beer Sheva 8443944, Israel

**Keywords:** lymphoma, Warthin tumor, parotid gland, parotidectomy

## Abstract

*Introduction*: A Warthin tumor is a benign salivary gland neoplasm, mostly found in the parotid gland. The number of reported Warthin tumors has increased over the years due to better diagnostic modalities and health system modernization. Warthin tumor rarely transforms into a malignant tumor; in this work, we present all cases reported in the English literature of different types of lymphomas within Warthin tumors. In this case, we present a low-grade follicular lymphoma arising within a Warthin tumor. *Clinical report*: A 64-year-old man presented to an oral and maxillofacial surgery clinic with a growing right facial mass. The medical history was significant for stable angina pectoris, hypertension, hypercholesterolemia, obesity, and a 20-pack-year smoking history. Fine needle aspiration suggested a diagnosis of Warthin tumor. A contrast CT scan of the parotid gland demonstrated a 2.9 × 2.7 × 4.1 cm diameter mass. The patient underwent right superficial parotidectomy. Histological examination of the mass revealed a low-grade follicular lymphoma arising in a pre-existing Warthin tumor. The postoperative PET CT showed no distant disease, and bone marrow biopsy during hematologic evaluation confirmed Stage 1 low-grade follicular lymphoma. The patient received 24 Gy of VMAT radiation therapy to the right parotid gland and continued hematologic follow-up. *Conclusions*: Based on a literature review, this is one of the few well-documented cases reported of low-grade follicular lymphoma within a Warthin tumor. This case highlights the importance of the thorough evaluation and diagnosis of parotid masses. Furthermore, this case reopens the debate on the “wait and see” approach regarding Warthin tumors. Fine needle aspiration-based diagnosis should not be considered final, as some malignant characteristics can be missed if declining surgery.

## 1. Introduction

Warthin tumor (WT), also known as adenolymphoma, papillary lymphomatous cystadenoma, cystadenoma lymphomatosum, and epitheliolymphoid cyst, is the second most common benign tumor of the parotid gland (17% of the benign tumors of the salivary glands) [1,2]. WT usually presents as an asymptomatic, slow-growing mass, commonly affecting middle aged to elderly men (>50 years), especially smokers, and can appear bilaterally in some cases [3].

According to the 2022 World Health Organization classification of tumors, WT is an adenoma composed of a bilayered columnar and basaloid oncocytic epithelium, supported by a dense lymphoid stroma that forms multiple cysts with numerous papillae, accompanied by a proliferation of follicle-containing lymphoid tissue [1,4,5].

Warthin tumor is believed to arise from intraparotid lymph nodes, as evidenced by the CAM5.2 staining of the extrafollicular reticulum cells within its lymphoid stroma, a fact that is important for understanding its unique histogenesis, distinguishing it from other salivary gland tumors, and guiding appropriate diagnostic and therapeutic approaches [6].

The treatment of WT usually involves the surgical removal of the tumor, including enucleation, extracapsular dissection, or partial parotidectomy; some selected cases allow for active surveillance [3].

The malignant transformation of WT is very rare but not impossible. There are reports of WT transforming into various carcinomas, Hodgkin’s lymphoma, and non- Hodgkin’s lymphomas. Some correlation of transformation has been found between WT and mucoepidermoid carcinoma, most likely representing cases of Warthin-like mucoepidermoid carcinoma [7].

Follicular lymphoma (FL) is the second most common subtype of non-Hodgkin lymphoma, accounting for approximately 20–25% of all new non-Hodgkin lymphomas diagnosed in Western countries [8]. It is slightly more common in men than in women (1.2:1) with an average age of 60–65 at diagnosis [8]. FL is a B-cell lymphoma arising from the germinal center of a lymph node, with most cells being centrocytes and centroblasts presenting a follicular growth pattern. A characteristic t(14;18) (q32;q21) translocation is found and positive CD20, BCL2, BCL6, CD10 on immunohistochemistry is indicative of FL [8,9]. In most cases, FL follows an indolent course, as it is traditionally a low-grade lymphoma. Patients commonly present with an advanced stage of the disease at diagnosis but are often asymptomatic, except for lymphadenopathy. The often characteristic B symptoms are generally absent [10]. The overall survival (median) for the majority of patients is more than 20 years [11]. Diagnosis is made via the excisional biopsy of the affected lymph node or extranodal mass. On biopsy, lymphoma cells present as a mix of small- to medium-sized cells (centrocytes) and large cells (centroblasts). The more centroblasts are present, the more aggressive the tumor. The WHO grading system for FL specimens defines three grades, where grades 1–2 consist of mainly centrocytes and are the least aggressive, grade 3a consists of some centroblasts and is slightly more aggressive, and grade 3b consists of larger count of centroblasts and is considered aggressive [10], carrying a worse prognosis and requiring a more aggressive treatment approach.

The treatment options for FL can range from expectant management to chemo-immunotherapy, depending on the lymphoma’s stage and grade as well as on the patient’s symptoms and tumor burden.

As an indolent lymphoma, FL resembles a chronic disease with an undulating course throughout life. As such, it requires clinicians to consider the need for treatment versus its long-term adverse events; FL can be silent for many years without symptoms even in the absence of treatment [12]. When needed, treatment is effective and can lead to a remission but, at some point, patients eventually relapse [13].

Initial-stage FL (stage I) can be managed with radiotherapy alone; however, only 10% of patients present with an early stage of the disease [10].

With advanced-stage disease, the treatment options vary greatly depending on symptoms, prognostic index score (such as FLIPI [14]), tumor burden (measured, for example, using the GELF criteria [15]) and tumor grade on biopsy [10].

When patients are asymptomatic and present with low-grade disease on biopsy and a low tumor burden, even at an advanced stage of the disease, expectant management is still the standard, namely, a watch-and-wait strategy; treatment at this point has not shown a benefit in terms of overall survival compared to follow -up aloe [12,13].

The treatment of FL reached a turning point with the development of rituximab, a monoclonal antibody that targets the B-cell marker of CD20. The development of this medication has allowed for an 88.4% 5-year survival rate compared to 70% in the 1990s. The median survival rate of newly diagnosed FL is 15–20 years [8,9].

This clinical report presents a rare case of low-grade FL arising in pre-existing WT and a review of the literature on hybrid WT and lymphoma cases. This case is unique in that it lacks systemic disease, highlighting the importance of including lymphoma in the differential diagnosis of parotid masses. Pathologists should carefully evaluate lymphoid follicles in Warthin tumors due to the risk of lymphoma.

## 2. Case Report

A 64-year-old man was referred to the Oral and Maxillofacial Department at Barzilai University Medical Centre, with a known right parotid mass that showed prominent growth in recent months.

His past medical history was significant for angina pectoris, hypertension, hypercholesterolemia, and obesity. In addition, he had been smoking 10 cigarettes a day for 40 years. The patient’s family, environmental, and occupational history were unremarkable.

Clinical examination revealed a 3 cm, partially fixed, nontender mass of rubbery consistency in the upper pole of right parotid gland, anteriorly to the tragus. The patient denied recent trave, zoonotic contacts and any of the B symptoms. The remainder of the physical and neurological examination and all laboratory tests were within normal limits.

**Figure 1:**
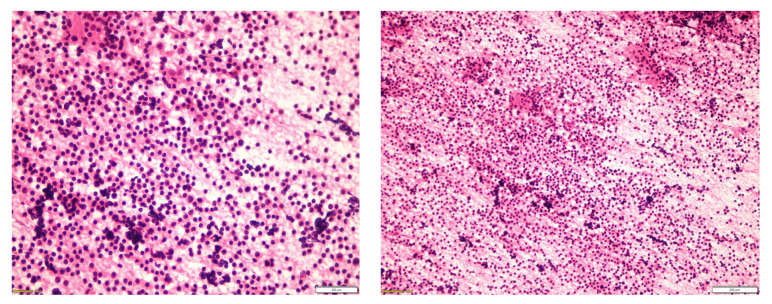
The typical cytomorphological features of the tumor are the cellular elements and cellular debris in the background, which consists of proteinaceous substrates. The cellular elements are scattered lymphoid cells and oncocytic cells. The oncocytic cells have abundant granular cytoplasm, round nuclei, and nucleoli.

Ultrasonography of the right parotid gland showed a hypoechoic lesion with an elongated liquid-tissue-like component, which demonstrated peripheral vascularity and measured 3.6 cm. Ultrasound-guided fine needle aspiration biopsy from the center of the lesion was performed, and the cytologic report suggested WT (Figure 1).

**Figure 2:**
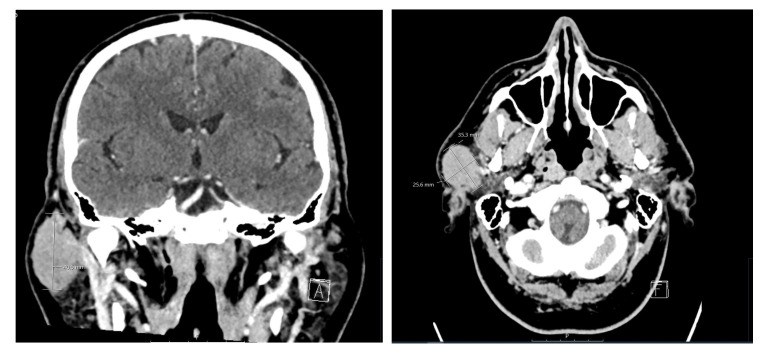
Preoperative coronal and axial views of CT imaging of the head and neck region. Soft tissue demonstrates an enhanced mass within the right parotid gland measuring at 2.5 × 3.5 cm. No enlarged lymph nodes or abdominal fluid collection can be seen.

Surgical removal of the tumor was recommended, and the patient was referred for a contrast CT for optimal surgical planning. A 2.9 × 2.7 × 4.1 cm diameter mass was detected in the superficial lobe of the right parotid gland. The mass was of solid appearance and demonstrated peripheral enhancement with contrast (Figure 2). No other cervical lymphadenopathy was noted.

The pathological and radiographic findings were discussed with the patient, and surgical excision was recommended and subsequently consented to by the patient.

**Figure 3:**
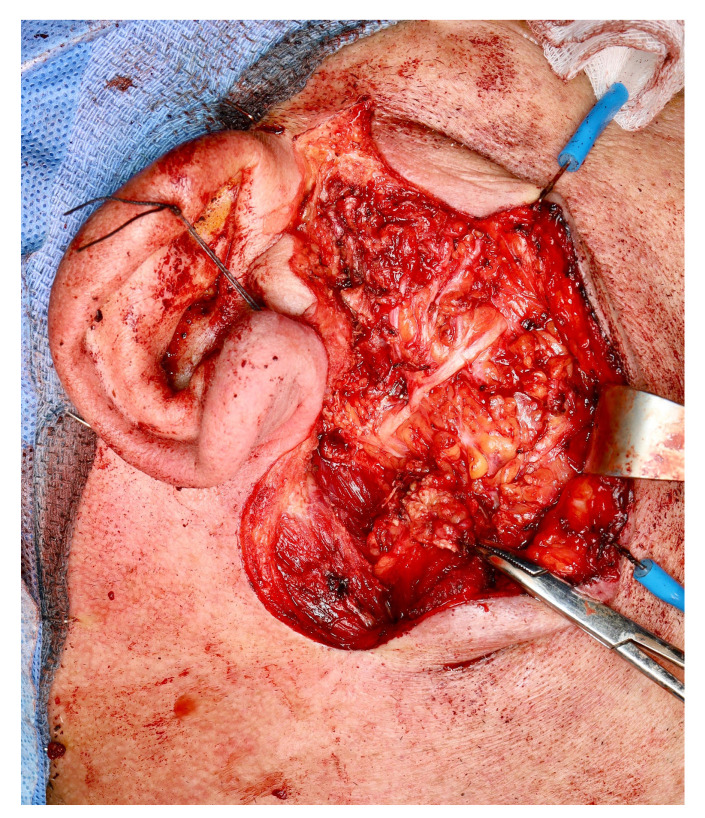
Intraoperative view of a surgical bed showing the facial nerve trunk and its upper branches. Tumor dissection in the area of a zygomatic branch was extremely challenging, involving significant mass adherence to nerve branches.

A superficial parotidectomy was performed. It is noteworthy that, during parotidectomy, the mass was adherent to the zygomatic branch of the facial nerve but was successfully bluntly dissected with no evident injury to the nerve (Figure 3). The excised tumor was of rubbery consistency with negative margins and a white-greyish appearance.

**Figure 4:**
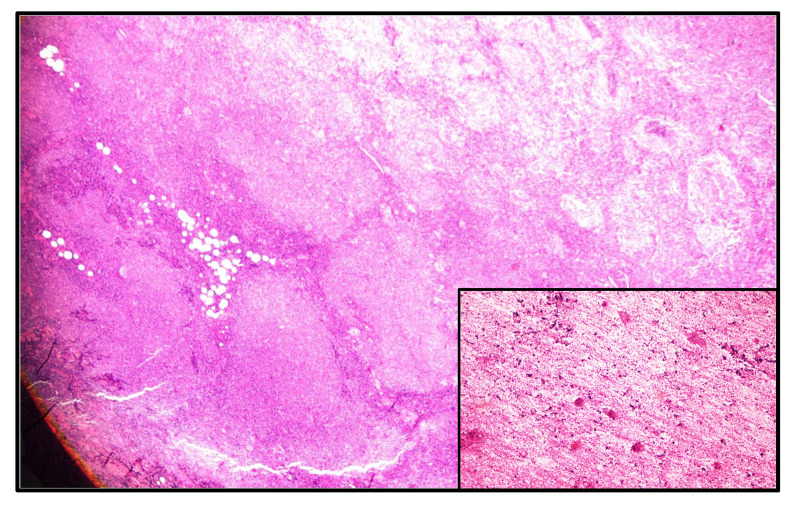
The photomicrograph presents packed neoplastic follicles with absent or attenuated mantle zones in an area previously diagnosed as Warthin tumor (inset from cytologic smear). No tangible body macrophages are noted. Most of the cells in the neoplastic follicles are centrocytes with scattered centroblasts < 15 per HPF.

On histological examination (Figure 4), the mass was consistent with low-grade (grades 1–2) follicular lymphoma arising in a pre-existing WT. Multiple sections from the macroscopically designated nodular mass showed low-grade lymphoma, composed of packed neoplastic follicles with absent and focally attenuated mantle zones and the absence of tangible body macrophages. Most of the cells in the neoplastic follicles were centrocytes, with scattered centroblasts (less than 15 per HPF). In addition, scattered oncocytic epithelial cells were present at the periphery of mass, suggesting a residual Warthin tumor. On immunohistochemical examination, the neoplastic lymphoid cells were positive for CD20, CD79a, BCL2, BCL6, and CD23. CD21 focally highlights follicular dendritic cells. CD3 and CD5 highlight interfollicular reactive T lymphocytes. Cyclin D-1 immunostaining was negative. Ki-67 was expressed in about 30% of lymphoma cells.

The PET CT showed postoperative hypermetabolic changes in the area of right parotid gland. Ipsilateral level IIa–IIB nodes were positive for FDG uptake.

The patient was referred for hematologic evaluation and continued follow-up, during which a bone marrow aspirate from the posterior left hip showed a normocellular appearance with no evidence of lymphoma invasion, confirming the diagnosis of Stage 1 low-grade follicular lymphoma. His treatment consisted of volumetric-modulated arc therapy (VMAT), delivered in 12 fractions of 2.0 Gy each, for a total dose of 24 Gy directed to the right parotid gland.

## 3. Review of the Literature

From a literature review, we found a total of 41 cases involving lymphoma in the parotid gland, as described in Table 1: 34 non-Hodgkin lymphomas and 7 Hodgkin lymphomas. Follicular lymphoma combined with WT was reported 15 times, with most cases being low-grade follicular lymphoma, as described in Table 2.

## 4. Discussion

In this case report, we present a 64-year-old man with a mass consisting of WT and low-grade follicular lymphoma. The incidence of WTs has increased over the years and accounts for 44.9% of parotid gland tumors according to a 42-year review from Franzen et al. [50]. This is a great increase compared to the previously known value of 12% from a 1977 report by Skolnik et al. [51]. New reports show evidence that WT has a clear predominance in the parotid gland over pleomorphic adenoma, making it the most common benign tumor of the parotid gland [50,52]. The male-to-female ratio decreased from 1:5.3 to 1:2.1, and this has probably been due to the advances in imaging techniques, health system availability, lifestyle changes, and the increase in smoking habits among female patients [50]. Malignant transformation of WT is extremely rare [3]. One of the first ever reported cases of a malignant tumor within a WT was from Ruebner and Bramhal in 1960, who presented a carcinoma arising in a WT [53]. According to the latest review of the literature published in 2020 by Alnoor et al., the prevalence of de novo lymphoma in WT was 3.4% [35]. Table 1 includes all the reported cases of WT with lymphoma in the English literature. Of the 41 cases, 34 were non-Hodgkin lymphoma (82.92%) and 7 were Hodgkin lymphoma (17.07%). A combination of follicular lymphoma with a WT is quite rare, with merely 15 reports in the literature including our report, most of them being low-grade follicular lymphoma [29,34,35,38,45]. From an embryological standpoint, lymphoma occurrence within the parotid gland is not surprising, since lymph nodes are encompassed by normal parotid tissue during fetal development. A lymphoma, thus, can potentially arise within the lymphoid stroma of a WT. This has been supported by several reports and indicates that lymphoma within a WT may remain localized for a long period of time [20,54].

Fine needle aspiration (FNA) biopsy has not proven to be an effective diagnostic modality when sampling the salivary glands. A false negative rate of up to 32% for malignant tumors has been reported for this technique in parotid gland pathology; thus, surgeons opt for the surgical removal of parotid gland tumors [55]. The clinical presentation of lymphoma in the parotid gland is clinically indistinguishable from other masses or lesions in the region. Consequently, lymphoma should be included in the differential diagnosis of all parotid gland swellings [56].

In our case, on the one hand, the FNA result showed that the tumor was classified as a MILAN IVa neoplasm with completely benign characteristics. On the other hand, there were some red flags, suggesting that was not an ordinary WT: the persistent growth, the rubbery consistency, and the ultrasound and CT that were not typical. In addition, during surgery, the dissection was not easy: the mass was hard to separate from the surrounding tissue, which is also not typical of WT.

The final pathology report of the superficial parotidectomy presented a completely different aspect, with a surprising finding of follicular lymphoma. This important finding may have been overlooked and missed had the patient declined surgery. This can also be explained because the diagnosis of FL is performed via excisional biopsy, as FNA should be avoided when trying to diagnose FL. The typical growth pattern and grading of the lymphoma cannot be determined with fine needle aspiration techniques, making it practically impossible for pathologists to classify the disease [9]. Some suggest core needle biopsy for salivary gland tumors [3], and, theoretically, a core biopsy in this case could have brought us to the correct diagnosis without need for surgery.

The current surgical approach for the removal of benign parotid tumors, extracapsular dissection, offers a less-invasive alternative to formal parotidectomy. Importantly, this technique is oncologically safe, results in functional outcomes, and has low postoperative complication rates, distinguishing it from the now obsolete practice of tumor enucleation. The widespread use of office ultrasound has made these tumors easier to diagnose and treat when indicated [57].

**Figure 5:**
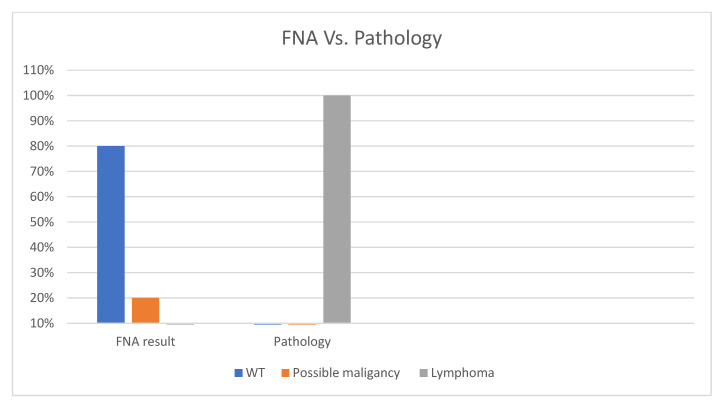
The difference between FNA and final pathology of the excised tumor. FNA results indicated that 80% of cases were WT, but the pathology of the tumor indicated 100% of the cases were lymphoma.

Out of the 41 cases of lymphoma combined with the WTs reported, FNA was performed in only 10 cases. Figure 5 demonstrates that 80% of these cases were mistakenly diagnosed as WT when FNA alone was performed; however, all of them were finally diagnosed as lymphoma when surgically removed and examined by a pathologist. These numbers emphasize the importance of thorough examination of all parotid gland specimens by a trained pathologist so that malignant tumors are not overlooked.

Our case presents clinical manifestations different from those of other follicular lymphomas within WT in the literature, mainly by showing no systemic disease. Thus, this entity should always be considered in a differential diagnosis and ruled out as the first presentation of systemic lymphoma, with full staging performed upon diagnosis. The cases of an initial finding of follicular lymphoma within a WT indicate that pathologists should take extra care when examining the lymphoid follicles of a WT due to the potential appearance of lymphoma in the WT [34].

Despite the low incidence of lymphoma in WT, this finding emphasizes the importance of conducting a thorough patient examination with a broad differential diagnosis. The widely accepted reliance on FNA techniques provides a fair contribution to diagnosis; however, the accuracy of parotid gland pathology leaves more room for proper clinical surgical judgement. Despite a reassuring FNA report, the surgical removal of tumors with adverse clinical features should be offered to determine final pathology.

## 5. Conclusions

This clinical report presented a low-grade follicular lymphoma arising within a WT. This extremely rare case emphasizes the need to follow good surgical clinical judgement as not all tumors appear in their classic form.

## Figures and Tables

**Table 1:** Characteristics of malignant lymphoma and WT.

Study and Year	Age	Sex	Site	FNA Result	Lymphoma Type
**Hodgkin Lymphoma**					
Melato et al. (1986) [16]	69	M	Right parotid	NA	CHL, mixed cellularity
Badve et al. (1993) [17]	76	M	Left parotid	NA	CHL, not distinguished
Cozzolino et al. (2009) [18]	60	M	Right parotid	WT	CHL, not distinguished
Ye-qing Liu et al. (2013) [19]	78	M	Left parotid	NA	CHL, lymphocyte-rich
Napoli et al. (2015) [20]	73	M	Left cervical mass	NA	NLPHL
Jun et al. (2018) [21]	59	M	Right neck mass	NA	CHL, mixed cellularity
Safi et al. (2018) [22]	80	M	Left parotid	NA	CHL, not distinguished
**Non-Hodgkin Lymphoma**					
Colby et al. (1979) [23]	NA	NA	Parotid	NA	Follicular lymphoma/grade 1
Seifert et al. (1980) [24]	83	M	Parotid	NA	Follicular lymphoma
Miller et al. (1982) [25]	49	M	Angle of mandible	NA	Follicular lymphoma/grade 1
Banik et al. (1984) [26]	75	M	Left parotid	NA	Follicular lymphoma/grade 2
	76	M	Right parotid	NA	Follicular lymphoma/grade 2
Hall et al. (1985) [27]	64	M	Right parotid	NA	Follicular lymphoma/grade 2
Griesser et al. (1986) [28]	64	F	Palate	NA	Follicular lymphoma/grade 2
Medeiros et al. (1990) [29]	71	M	Left parotid	NA	Follicular lymphoma/grade 2
Giardini et al. (1990) [30]	57	M	Right and left parotid	NA	Follicular lymphoma/grade 1
Shikhani et al. (1993) [31]	56	M	Right parotid	WT	Follicular lymphoma
Park et al. (2000) [32]	68	F	Right perparotid lymph node	NA	Follicular lymphoma/grade 1
	55	M	Right parotid	NA	Follicular lymphoma/grade 1
Romero et al. (2016) [33]	82	F	Right parotid	NA	Follicular lymphoma
Alnoor et al. (2020) [34]	69	M	Right neck swelling	WT	Follicular lymphoma/grade 1–2
Alnoor et al. (2020) [35]	74	M	Left parotid (bilateral on CT)	WT	In situ follicular neoplasia
Current case	64	M	Right parotid	WT	Follicular lymphoma/grade 1–2
Reiner et al. (1979) [36]	56	M	Right parotid	NA	DLBCL
Griesser et al. (1986) [28]	82	M	Left submandibular	NA	DLBCL
Gorai et al. (2007) [37]	102	M	Left neck mass	Class II or no malignancy	DLBCL
Ozkok et al. (2012) [38]	60	M	Left side of the jaw, swelling	NA	DLBCL
Chu et al. (2015) [39]	83	M	Left parotid	NA	EBV-positive DLBCL
Wang et al. (2019) [40]	67	M	Right parotid	NA	DLBCL
Gutierrez-Alvarez (2023) [41]	65	M	Right parotid	NA	DLBCL
Bunker et al. (1989) [42]	63	F	Left parotid	NA	SLL/CLL
Saxena et al. (2005) [43]	60	M	Left parotid	NA	SLL/CLL
Jawad et al. (2018) [44]	80	M	Right parotid	Small lymphocytes admixed with occasional larger lymphoid cells	SLL/CLL
Alnoor et al. (2020) [35]	60	M	Right parotid	WT	SLL/CLL
Seifert et al. (1980) [24]	71	M	Parotid	NA	Mantle-cell lymphoma
Arcega et al. (2015) [45]	70	M	Left neck mass	NA	Mantle-cell lymphoma
Marioni et al. (2004) [46]	61	F	Right parotid	WT	MALT-type lymphoma
Pescarmona et al. (2005) [47]	66	M	Right cervical lymphadenopathy	WT	Nodal peripheral T-cell lymphoma, NOS
Giaslakiotis et al. (2009) [48]	81	M	Right parotid	NA	T-cell lymphoblastic lymphoma
Pan et al. (2019) [49]	69	F	Left side of neck	NA	T-cell lymphoblastic lymphoma
Colby et al. (1979) [23]	52	M	Parotid	NA	Lymphoma, unclassified

**Table 2:** Summary of all reported cases involving WT and lymphoma.

**WT and Lymphoma**	***N*** **= 41**
**Hodgkin Lymphoma (n = 7, 17.07%)**	**Frequency**
CHL, mixed cellularity	2 (31, 35)
CHL, lymphocyte-rich	1 (34)
CHL, not distinguished	3 (32, 33, 36)
NLPHL	1 (27)
**Hodgkin Lymphoma (n = 34, 82.92%)**	**Frequency**
Follicular lymphoma	15 (22, 25, 37–46)
In situ follicular neoplasia	1 (21)
DLBCL	7 (23, 42, 47–51)
SLL/CLL	4 (21, 52–54)
Mantle-cell lymphoma	2 (38, 24)
MALT-type lymphoma	1 (55)
Peripheral T-cell lymphoma	1 (56)
T-cell lymphoblastic lymphoma	2 (57–58)
Unclassified	1 (37)

## Data Availability

Data are contained within the article.
